# General Medicine and Therapeutics

**Published:** 1895-07

**Authors:** 


### GENERAL MEDICINE AND THERAPEUTICS.

EDITED BY DR. LUTHER B. GRANDY.

### THE TREATMENT OF MALARIAL HEMATURIA.

The *Therapeutic Gazette* for May publishes the results of a collective investigation undertaken by the editor, Dr. Hare, on the treatment of malarial hematuria. He sent sets of questions to a large number of physicians in parts of the country where this disease seems to be indigenous—Alabama, Georgia, Mississippi and Texas. Replies from 107 show the following remedies to be in use: Calomel (33 physicians), tincture of iron (25), arsenic (23), ergot (18), turpentine (14), sodium hyposulphite (10), morphine (9), gallic acid (7), nitrate of potash (5), mineral acids (9), etc.

Following is a summary of the views expressed by these physicians:

*Calomel* is used by thirty-three physicians who have reported to us, of whom two are from Alabama, four from Georgia, seventeen from Mississippi, and ten from Texas.

Moody, of Alabama, gives it in large doses (five grains every hour) until tarry discharges.

Cheatham, of Georgia, finds that when he gets good action from calomel, patients do well.

McGehee, of Mississippi, regards calomel as the "sheet-anchor."

Weight, of Mississippi, who has had an experience of one hundred and thirteen cases of malarial hematuria, gives fifty grains of calomel every three hours until three doses have been taken; has never seen a case ptyalized by this large dose. Whether the drug acts by relieving and stimulating the hepatic gland, and preparing for or aiding the action of quinine, or whether its action in stimulating the flow of bile and urine with increased elimination of urea produces the desired result is difficult to decide.

Price, of Mississippi, in "alterative doses."

Kendrick, of Mississippi, regards calomel as the "sheet-anchor."

Ford, of Mississippi, has more confidence in it than in any other remedy.

McPheeters, of Mississippi, uses it at onset with sodii bicarb., of each five grains.

Dunn, of Mississippi, administers ten to twenty grains, in five-grain powders, every one or two hours until dark biliary action.

Lester, of Mississippi, gives thirty grains, repeated every two hours, until three or four doses are taken.

Smith, of Texas, advises calomel above all else.

*Tincture of Chloride of Iron.*—Guice, of Mississippi, considers tr. ferri chlor. combined with arsenous acid the remedy *par excellence.*

Lester, of Mississippi, considers the diseased condition of the red corpuscles and administers iron.

Brownrigg, of Mississippi, and MacKenzie, of Texas, give the tincture of iron chloride (twenty drops every four hours).

Shaw, of Texas, uses it alone or combined with small doses of quinine.

*Sodium Hyposulphite.*—Greene, of Mississippi, considers it as positively germicidal and as aiding in elimination.

Jones, of Mississippi, considers it the best remedy, combining it with gelsemium and ergot.

Dunn, of Mississippi, gives twenty to forty grains every three hours, after thorough purgation with calomel.

Eliot, of Mississippi, considers it among the best remedies after quinine.

*Ergot.*—Guice, of Mississippi, uses ergot hypodermically only as an adjunct to turpentine.

The majority of those using it regard it as useful as a hemostatic.

*Arsenic.*—Moody, of Alabama, administers liquor potassii arsenatis (one drop every half-hour until rigors are overcome).

Guice, of Mississippi, uses arsenous acid as a substitute for quinine early and throughout the attack; also for the relief of malarial cachexia, considering it the remedy *par excellence.*

Dunn, of Mississippi, uses it only after the urine has become clear, in the dose of four to five drops t. d. of Fowler's solution.

Montgomery, of Mississippi, gives five drops of Fowler's solution every two hours.

Twenty-three other physicians also recommend it.

*Morphine.*—Robertson, of Alabama, uses morphine "to quiet restlessness and reduce fever."

Smith, of Mississippi, uses it to relieve pain.

Brownrigg, of Mississippi, keeps patient constantly under influence of morphine, while Stockard uses it continuously "only for sedation."

McAdams, of Texas, uses it combined with belladonna hypodermically.

Swearington, of Texas, uses it in small doses..

Conalley, of Texas, gives one-sixth to one-fourth grain hypodermically, if much nausea or high temperature is present.

Watkins, of Texas, treats cases of hematuria with quinine combined with morphine "to quiet the stomach."

*Turpentine.*—Guice, of Mississippi, says: "To arrest the renal hemorrhage I have found turpentine the best agent in the dose of ten drops every four hours, given in capsules, using at the same time turpentine liniment on the lumbar region.

McPheeters, of Mississippi, also gives same dose every three hours until the urine clears; if the stomach proves irritable he administers it by enema.

Lester, of Mississippi, values turpentine, thinking it aids in warding off uremic poisoning. He has had one hundred and twenty cases.

*Gallic Acid.*—Moody, of Alabama, uses tannic or gallic acid in small doses, before nausea occurs, giving one-half grain at intervals of thirty minutes.

Kelly, of Georgia; Price, of Mississippi; Daily, Burts, and Oliver, of Texas, use tannic acid for the hemorrhage. Oliver combines it with calomel and sodium bicarbonate, as follows:

℞ Hyd. chlor. mite. ........................................................gr. xvi.
Sodii bicarb.,
Tannic acid, of each........................................................ gr. viii.
M. ft. charta, no. iv.

*Potassium Citrate.*—Moody, of Alabama, thinks potassium citrate, prepared by saturating strong lemonade with potassium bicarbonate, is beneficial in allaying nausea and increasing secretion of urine.

It is also recommended by Cochran, of Alabama.

*Potassium nitrate* is used by Fort, of Texas, for diuretic effects.

Davis, of Texas, administers it in thirty-grain doses.

The reader is referred to a former paper by Dr. Hare on quinine in malarial hematuria—*Therapeutic Gazette*, July, 1892.

---

### TREATMENT OF BRIGHT'S DISEASE.

General hygiene and the hygiene of the alimentary tract are considered by Sapelier (*Bull. Gén. de Thér.*, November 30, 1894) to constitute the chief and best therapeutic measures in the treatment of this disease. Medicaments are only of secondary or occasional importance. This treatment must be insisted on with great severity, and the patient's friends and family should be made aware of the fact that much depends on the strict observance of the rules laid down.

*General Hygiene.*—Whenever there are marked acute symptoms the patient should be put to bed. The patient should sleep only between woollen or flannel sheets. It is uncomfortable, perhaps, for a day or two, but the patient soon becomes accustomed to it. When dyspnea prevents rest in bed, great care should be taken to prevent the patient from taking cold. Woollen should be always next to the skin of the patient.

Southern climates are preferable in winter, with great care not to expose themselves during the mornings and evenings; while in the summer they should not visit the seashore. Woollen next to the skin not only prevents taking cold, but also slightly stimulates the action of the skin. This stimulation may be increased by the use of frictions with gloves made of horse-hair, which the author advises to be given every morning over the whole body.

Warm baths and vapor baths are to be avoided, as the chances of chills are very great. If rest in bed is not indicated, and there is no œdema to prevent walking, the patient should take moderate exercise, but not sufficient to fatigue. The author would prohibit the use of the bicycle in all cases. Walking and riding are the only forms of exercise. Where these are not possible, massage should be employed.

Marriage should be prohibited in women, or, at least, pregnancy in those having Bright's disease, and moderation in the sexual life of men. Tobacco is also an enemy of patients suffering with this disease; if it does not act directly upon the kidneys, it acts upon the heart, already menaced.

*Alimentary Hygiene.*—Milk is the food and medicine *par excellence* in Bright's disease. It should constitute the sole food during acute exacerbations and the principal drink at all other times. The amount taken should not exceed two and a half quarts per day; it can be either boiled or not, hot or cold, as the patient prefers. It should be taken in small quantities every two hours, slowly in swallows, to break up as much as possible the curds formed in the stomach. If necessary, instead of cow's milk, as usually employed, ass's or mare's millk, or kumyss, may be substituted; or a little tea or coffee to flaver may be added. The addition of stimulants should be avoided; while certain aromatics, as orange-flower water, may be employed. If the digestion of the milk is difficult, Vichy may be employed, or the following may be made use of:

Tr. nucis vomicæ,

Tr. star anise, of each............................gtt. xv to xx.

Syr. simplicis....................................f℥ iss.

Aq.................................................f℥ iii.

M. Sig.—One tablespoonful with each portion of milk.

And at the same time a pill of pancreatin.

In short, it should be the effort of the physician to impress upon the patient, and those about him, the fact that milk is the foundation *sine qua non* of treatment; that without it nothing can be accomplished. The diet should be confined as nearly as possible to this. They may be allowed, however, cream, egg-flips, soups with bread or vermicelli, or with meal, prepared with milk, fresh cheese, a little stale bread or crackers. On the first symptom of a relapse, immediate return should be made to a milk diet; this is the one rule that should govern the hygiene of the patient suffering from Bright's disease.

When the symptoms of disease have been absent for some time, a vegetarian diet may be inaugurated. The regimen may then include purées of all farinaceous materials, all doughs used as food, all flours or artificial mixtures of flours, all green vegetables prepared with butter or milk, eggs (preferably the yolks), fruits (preferably cooked), bread and crackers. The drink should be preferably milk, about two to three quarts per day. If milk cannot be taken, pure water or hot aromatic drinks alone are allowed—tea or Paraguay tea.

When even the menace of subacute attacks no longer exists,—that is, a relative condition of health has been obtained,—the diet may be made more liberal yet by the addition to the vegetarian diet of the white meats. Fresh pork is most easily tolerated by the diabetic patient, while veal is the least easily tolerated.

These are the substances which may be tolerated in the diet of the nephritic patient. Let us consider those that must be avoided. They are stale or fermented cheese, black and red meats, game, all forms of pork other than fresh pork, bouillon (a veritable solution of ptomaïnes), all meat juices and extracts, all forms of fish and of shell-fish.

All these foods are dangerous, either on account of their nature, the putrefaction more or less advanced, which easily may escape detection either by taste or smell, and they always contain a more or less large quantity of toxins. These toxins which the kidneys cannot eliminate are the constant danger of the nephritic patient; they should not be taken in if it be possible to avoid them.

Alcohol is universally condemned in these cases; beer is useful; some physicians permit the use of wine in the periods between attacks; it is not, however, necessary to life and should be avoided. Liquors and all strong alcoholics should be probibited.

*Pharmaceutical Therapeutics.*—Of all the drugs which have been used in this disease the author would retain digitalis, caffeine, theobromine, and perhaps the iodides, with the addition of the revulsives and venesection.

The duty of the physician in these cases, after prescribing the milk diet, is to relieve the system of its toxins by the administration of a purgative. The activity of the purgative should depend on the necessity for immediate action, or, if the symptoms are not marked, milder purgation may be employed. Whenever the acute or subacute symptoms reappear, purgation should again be resorted to. Salines the author has found are often not well tolerated, and he prefers to use scammony in capsules, in doses of seven to nine grains, or even, when the case demands, as high as fifteen grains. It is easily taken without being tasted, and has but a slight tendency to produce cramps, and he considers it the preferable purgative in these cases.

At the same time that relief is sought through the intestine, counter-irritation should be applied in the lumbar region over the kidneys, in the form of sinapisms, wet or dry cups, or superficial touches with the actual cautery. Vesicants and the tincture of iodine should be avoided.

In case of acute symptoms, as uremia of a nervous type, acute delirium, epileptiform convulsions, coma, violent dyspnea, in addition to these measures, bleeding should be used. By the removal of ten to fourteen ounces a notable quantity of uremic poison is removed. Applied properly in certain cases, bleeding renders invaluable relief to diabetics, and it should not be forgotten.

If the heart is weakened, besides the measures already mentioned, the hypodermic injection of caffeine is indicated. The following solution should be employed:

℞ Caffeine.

Sodii benzoat., of each......................................gr. lxxv.

Aquæ..................................................f℥ vss.

M. Sig.—Inject subcutaneously 15 minims,—that is, each injection contains 4 grains of caffeine.

The absorption is much more rapid and less irritant by this method than when taken into the stomach. If there is œdema, the heart is dilated, the pulse small and weak, digitalis is indicated.

Except in cases of acute attack, the entire treatment should be hygienic. In the case of patients who have, in addition an arteriosclerosis, the iodide of sodium, in a daily dose of seven to fifteen grains, should be taken for three weeks of each month.

In closing, the author reiterates that *milk* is the most certain and inoffensive of diuretics in these cases.—*Therapeutic Gazette.*

---

### ANEMIA IN CHILDREN.

The paper deals with simple anemia only. In this condition the proportion of hemoglobin is diminished to a much greater extent than is the red blood-globules. Chief among the various causes of the condition are malnutrition secondary to graver disorders, improper and scanty food, faulty hygiene, and a condition of uricacidemia. The author agrees with Rachford that pronounced anemia without apparent cause is strongly suggestive of concealed tuberculosis. The condition is frequently due to deep-seated and hidden glandular tuberculosis.

In simple anemia the hemoglobin is reduced; the red blood-cells may or may not be diminished in number; the total bulk of blood may or may not remain the same. The initial symptom is therefore pallor, the color ranging all the way from simple whiteness to dusky yellow. In many cases this is shown chiefly in the lips, gums, and roof of the mouth. The pulse is frequently full and soft, the heart's action irregular. A venous hum is commonly heard over the jugulars.

Diagnosis must be made from chlorosis, pernicious anemia, leukemia, beginning tuberculosis, and acute Bright's disease. In chlorosis, which is extremely rare in young children, the color is characteristic. In pernicious anemia the red blood-corpuscles are greatly decreased in number. The white corpuscles are also somewhat diminished. In leukemia, a very rare disease in young children, there is a steady decrease in the number of red blood-cells, with a constant and steady increase in the number of white cells; the liver, spleen, and lymphatic glands are enlarged. In the management of anemia fresh air, regular exercise, and fatty food are especially indicated. The two drugs which are most efficacious in the treatment of anemia in young children are iron and arsenic, which should be given sparingly in tonic doses. In those forms of anemia which are more or less clearly intestinal antiseptics should be administered.—DR. WARNER, *Med. Record.*

---

### THE TRUTH ABOUT ALCOHOL AND TOBACCO.

Dr. Albert R. Ledoux, in a recent paper before the New York Society of Medical Jurisprudence, treats of "Popular Fallacies as to Alcohol and Tobacco," and makes clear the mischief which may follow a confusion of ideas with respect to pure and impure liquors, tobacco drugged and undrugged. He scores a good point when he reminds us that a man may become a drunkard from pure as well as impure whisky, and that it is folly to blame the adulterant for the evils wrought by the alcohol.

Cigarettes are abominable, not on account of their adulterants—for opium and arsenic are too expensive to be incorporated into cigarette tobacco—but because they appeal to immature consumers, and more nicotine is absorbed when the body is growing. The mild and pleasant flavor is likewise alluring and conduces to habitual inhaling. Dr. Ledoux's inferences are sound and have the merit of so vigorous an expression as to merit quoting:

"The purest tobacco is undoubtedly that which is prepared for pipe-smoking—purer, in my opinion, than that found in the average cigar; yet the nearer it comes to the absolutely pure leaf the higher the per cent. of nicotine. But neither in the cigar, nor cigarette, nor chopped or cut tobacco is there at this day, among all the adulterations, anything approaching in potency this nicotine which they contain.

"Whether we are smokers or non-smokers, whether we are moderate or immoderate drinkers, or total abstainers, let us fearlessly and honestly and intelligently instruct the rising generation that alcohol and tobacco are substances to be avoided by youth, no matter in what form or under what name they may be sold; and let the intelligent physician, who meets in his practice the too slavish devotee of the tobacco habit or the votary of alcohol, inform his patient, in all candor and seriousness, that it is alcohol and nicotine that he must let alone, and not endeavor to shift him from port to sherry, or from cigar to pipe, under the vain delusion that if one harms the other will benefit."—*Bulletin of Pharmacy.*

---

### COMMON MEMBRANOUS SORE THROAT.

The correctness of the position taken by Professor J. Solis-Cohen, Sir Morell Mackenzie and other authors as to the existence of *membranous sore throat, not diphtheric in character,* has come to be universally admitted, and the claims advanced for certain methods of treatment in diphtheria proved to be based upon erroneous diagnosis. A recent illustration of this fact has come under notice at the Laboratory of Bacteriology, where culture from three cases of membranous sore throat much resembling diphtheria in appearance, in two of which there was high fever accompanied with constitutional depression, proved to be free from Klebs-Loeffler bacilli.—*Med. and Surg. Reporter.*

